# Exploring the Adherence to the Mediterranean Diet and Its Relationship with Individual Lifestyle: The Role of Healthy Behaviors, Pro-Environmental Behaviors, Income, and Education

**DOI:** 10.3390/nu10020141

**Published:** 2018-01-28

**Authors:** Alessia Cavaliere, Elisa De Marchi, Alessandro Banterle

**Affiliations:** Department of Environmental Science and Policy, University of Milan, via G. Celoria 2, Milano 20133, Italy; alessia.cavaliere@unimi.it (A.C.); alessandro.banterle@unimi.it (A.B.)

**Keywords:** Mediterranean diet, sustainable diets, healthy behaviors, pro-environmental behaviors, consumer behavior, structural equation modeling

## Abstract

The reduction of diet-related diseases and the improvement of environmental sustainability represent two of the major 21st century food policy challenges. Sustainable diets could significantly contribute to achieving both of these goals, improving consumer health and reducing the environmental impact of food production and consumption. The Mediterranean diet (MD) represents an excellent example of sustainable diet, however recent evidence indicates that such a dietary pattern is now progressively disappearing in Mediterranean countries. In such a context, this paper explores how individual lifestyle and habits are related to a high/low adherence to the MD model. The goal is to examine whether there is a relationship between individuals’ healthy and pro-environmental behaviors and their level of adherence to the MD. The analysis also explores the role of consumer income and education. The study is based on the Italian population (*n* = 42,000) and uses a structural equation model approach. The results outline that the MD is part of a sustainability-oriented lifestyle and stress the key role of both income and education in affecting adherence to MD. Future policy aimed at contrasting the gradual disappearance of the MD should emphasize the sustainable dimension of the MD, meanwhile reducing socio-economic disparities among different population segments.

## 1. Introduction

Improving public health by reducing the prevalence of diet-related diseases and increasing environmental sustainability represent two of the major 21st century food policy challenges. Overweight and obesity are constantly increasing both in developed and developing countries, and the dramatic increase in such diet-related diseases, besides representing a public health concern, has a negative impact on economic systems generating considerable costs. According to the World Health Organization, in 2016 more than 1.9 billion adults were overweight and over 650 million of these were obese [1]. These alarming data reflect the overall inadequacy of current food consumption patterns and highlight the need to pursue healthier dietary habits. Meanwhile, food consumption needs to become more environmentally sustainable in order to safeguard biodiversity of ecosystems and guarantee the wellbeing of future generations. Previous studies estimated that the agri-food system impacts considerably on the environment, accounting for almost 70% of freshwater and 20% of energy use, and contributing dramatically to greenhouse gas emissions and land use [2,3,4]. 

The reduction of diet-related diseases and the improvement of environmental sustainability represent two goals with one common denominator, diet. The strong link between health and the environmental dimensions of food consumption is well emphasized in the concept of ‘sustainable diet’. This notion was firstly introduced by Gussow and Clancy in the early 80s, to refer to dietary patterns that are healthier for both the environment and the consumers. More recently, the Food and Agriculture Organization (FAO) extended and formalized the concept of sustainable diet, stating that: “Sustainable diets are those diets with low environmental impacts which contribute to food and nutrition security and to healthy life for present and future generations”. Additionally, FAO reports that sustainable diets are “nutritionally adequate, safe and healthy; while optimizing natural and human resources” [5].

An excellent example of sustainable diet is represented by the Mediterranean diet (MD), characterized by a nutritional model based on the consumption of low processed food, mainly cereals, and fresh or dried fruit and vegetables, with a moderate consumption of fish, meat, and dairy products, and the use of olive oil as the main condiment [6,7]. The MD has been amply recognized in the past to have a preventive effect against excess weight gain, cardiovascular diseases, diabetes, and even some types of cancer [8]. In addition to its proven contribution to the maintenance of a good health condition, the MD has a low environmental impact. Indeed, according to this dietary model the food categories that must be consumed more frequently to guarantee nutritional adequacy (namely, plant-based foods), are also those foods whose production and consumption have a lower impact on the environment [9,10]. Moreover, the importance of the MD as a model of sustainable diet does not only lie in its specific nutritional recommendation, but also in its core philosophy of sustainability that involves the protection of biodiversity, local production, and cultures in respect of the beliefs of each community [6].

Despite these anticipated benefits, recent evidence indicates that the MD is now progressively disappearing in Mediterranean countries, particularly amongst the youngest generations [6,11]. This has been partly attributed to the rapid and widespread dissemination of Western-type dietary models and to food globalization [6]. Some studies also examined the specific role of taste, convenience, and price in determining a deviation from the MD [12,13,14,15]. This paper adds to this field of research by exploring the relationship between some aspects of individual lifestyle and socio-economic status and the level of adherence to the MD. In detail, this paper investigates whether there is a relationship between the extent to which individuals engage in healthy and pro-environmental behaviors and their compliance with the MD guidelines. In addition, based on recent evidence stressing the crucial role of income and education in affecting food choices, the analysis investigates the role of these two factors in affecting the extent to which individuals follow the MD guidelines. The goal is to contribute to identifying the main factors that can influence individual adherence to the MD in order to provide novel insight that could be used to formulate specific food policy interventions geared at promoting the MD model. Indeed, the gradual disappearance of the MD heritage that many Mediterranean countries are experiencing is alarming for several reasons, which includes the long-term negative impact on people’s health and on the environment, alongside significant negative effects on the social, cultural, and economic context of the Mediterranean regions [16]. To this purpose, the study uses a structural equation model performed using the data of the Italian Household Survey provided by the Italian National Institute of Statistics which includes 42,000 observations.

The present paper is organized as follows: the following subsections provide the literature background on healthy and pro-environmental behaviors and illustrate the theoretical framework; Section 2 describes the sample population and explains the approach to the analysis and methodology applied; Section 3 describes the main results; and Section 4 provides the discussion and conclusion.

### 1.1. Healthy and Pro-Environmental Behaviors: Background and Hypotheses

According to previous literature, healthy behaviors (HB) relate to manner of behaving that is clearly oriented to health. They can be described as all behaviors and activities aimed at maintaining or improving the health condition [17,18]. Therefore, by definition HB include a number of different activities that can exert a direct (e.g., exercise and non-smoking) or an indirect (e.g., information seeking) positive effect on health [19].

Similarly, pro-environmental behaviors (PEB) refer to those personal actions that are directly related to environmental improvements [20] and include all activities that “consciously seek to minimize the negative impact of one’s actions on the natural and built world” [21].

HB and PEB differ in many respects as the former are mainly related to individual private utility (being in good health), while the latter are typically motivated by a social concern and are mainly linked to social utility (well-being of future generations) [22,23,24]. However, previous studies found that HB and PEB are both guided by individual future orientation. Future oriented individuals, tend to value future outcomes of present actions to a greater extent compared to present oriented people. This characteristic affects their lifestyle, making them more prone to sacrifice immediate gratification in order to obtain future benefits. As a result, these individuals are typically more willing to engage in health enhancing and environmentally sustainable behaviors [17,18,23,25]. Accordingly, it is reasonable to expect that these individuals are more prone to follow the MD guidelines. Indeed, there are at least four sustainability benefits that could be derived from the MD. Two of them, respectively represented by major health and nutrition benefits, are related to the health domain; the other two are connected to the environment dimension and are respectively represented by the low environmental impact and richness in biodiversity [11]. As such, the first hypotheses tested in this work are the following:

**Hypothesis 1** **(H1).** There is a positive relationship between HB and individual level of adherence to the MD.

**Hypothesis 2** **(H2).** There is a positive relationship between PEB and individual level of adherence to the MD.

### 1.2. Education and Income: Background and Hypotheses

According to previous studies diet quality varies according to a socioeconomic gradient such that disadvantaged groups are more likely to have poor dietary patterns [26]. This is mainly attributable to low education and income that negatively impact on individual dietary choices. As demonstrated in previous works low education is typically associated with scarce nutritional knowledge and low awareness regarding food-related issues. In these cases, consumers may fail to follow nutritional recommendations and guidelines for a healthy diet [27,28].

Low income, instead, affects the type of food products that consumers buy both in terms of quality and variety. Such evidence is supported by the results of a recent cohort-based epidemiological study conducted on the Italian population. The study examined the specific relationship between education and income with adherence to the MD providing evidence that the socioeconomic status (SES) has a crucial role [13,29]. The authors found that the protective effect of the MD against cardiovascular disease risk is evident among the highest SES groups, with high education high income, whilst no relationship was found for less educated groups in the lower income categories [13,29,30]. The study, indeed, outlined that individuals in high SES groups tend to consume whole grain cereals and fish more frequently, meat in low amounts, and to have a higher diet variety with regard to vegetables.

Accordingly, it is hypothesized that:

**Hypothesis 3** **(H3).** High income is associated with higher adherence to MD.

**Hypothesis 4** **(H4).** High education is associated with higher adherence to MD.

## 2. Methods

### 2.1. Study Population

The analysis is based on the data collected through the Italian Household Survey conducted in 2012 by the Italian National Institute of Statistics (Istat). The Italian Household Survey involves a large sample that covers the resident population by interviewing a sample of 20,000 households corresponding to 42,000 individuals. The survey includes questions about peoples’ habits (including food habits), lifestyle, socio-demographic data, and socio-economic status. The Household Survey sample is selected following a multistage random sampling procedure based on the population census. Italian cities are stratified on the bases of the demographic dimension and other criteria. A few days before the interview, all sampled households were informed about details of the survey through a letter from Istat and the interviews were taken in the presence of an expert interviewer. The questionnaire was anonymous and included items with a multiple choice format response or with rating scales (dummy and ordinal variables). A total 38, items were considered for the purpose of this analysis.

### 2.2. Approach to the Analysis

The data were analyzed using SPSS (version 24 SPSS, IBM, New York, NY, USA) for the descriptive statistics (e.g., variable mean, standard deviation, etc.) and AMOS to perform the Structural Equation Modeling (SEM). SEM is a multivariate statistical method combining factor analysis and multiple regression analysis and is used in social and behavioral sciences to test theoretically supported structural relationships. This method overcomes the main limitations of the classic regression models in that it allows to simultaneously estimate multiple and interrelated relationships among dependent and independent variables [31,32].

After elaborating the theoretical framework based on the hypotheses specified in Section 1.1 and Section 1.2 we followed two main steps. Firstly, we defined the latent variables respectively expressed by multiple measured variables (i.e., indicators) grouped through a confirmatory factor analysis (factor loadings data available upon request). Secondly, we applied the structural equation modeling approach based on the estimation of two sub-models. The first is the measurement model (or inner model), which is referred to the relationships between the dependent latent variable and the independent latent variables. In our model, the dependent latent construct is represented by the individual adherence to MD, while the independent latent constructs are HB, PEB, income, and education. All details on the measured variables used to generate the latent constructs are provided in the following sections. The second sub-model is the structural model (or outer model), which refers to the relationships between the latent variables and their indicators. In our study, the relationships between the latent variables and the indicators were defined through a reflective measure specification, where the latent constructs are assumed to exist independently of the indicators used and variation in the measured variables do not cause variations in the construct. Each manifest variable is assumed to be a linear function of its latent variable and residual. Moreover, in reflective models, adding or dropping an item does not change the conceptual domain of the construct [30]. The outer relationships depend on the predictor specifications and it is assumed that no correlations exist between outer residuals and the related latent variable.

Before the model testing, we performed all the descriptive statistics and checked for internal consistency of the relevant variables accounting for missing data. As a final step, we examined the structural model validity.

### 2.3. Measures

#### 2.3.1. Adherence to the MD

The first latent construct of the model is represented by a measure of individual adherence to the MD described by three indicators. The three indicators are respectively represented by three indexes: the food consumption index, the fat and salt consumption index, and the drink consumption index, each one comprising several items.

The food consumption index (MD1) was constructed based on 16 questions included in the Household Survey regarding the consumption frequency of different food items covering all the food groups of the MD pyramid [33,34]. The 16 questions were presented in the questionnaire in a multiple choice format with five response alternatives (single response allowed), as illustrated in Table 1. To construct the MD1 index individual responses were recoded to reflect the degree of adherence to the MD assigning scores ranging from 0 to 2, where 0 indicates low adherence, and 2 high adherence. The scoring criteria were based on the MD pyramid guidelines for adult population and the resulting individual food consumption index ranged from 0 (minimum adherence to the MD) to 32 (maximum adherence to the MD).

As for the fat and salt consumption index (MD2), we used two questions related to the main oils and fats used for cooking and seasoning respectively. Value 2 was assigned when respondents choose olive oil as the main condiment used for cooking and seasoning, value 1 was assigned to ‘other vegetable oils and fats’, and value 0 to ‘animal fats’. With regard to salt, the scoring assigned value 2 when respondents responded ‘I am very careful about the quantity of salt used’, value 2 was assigned to ‘I have progressively reduced the quantity of salt used’, whereas value 0 corresponded to ‘I do not pay attention to the quantity of salt used’.

The drink consumption index (MD3) was constructed based on four questions referred to the consumption frequency of water, beer, wine, and soft drinks. In this case, the Household Survey provided the following response alternatives: ‘Zero consumption’, ‘Seasonal consumption’, ‘Rarely’, ‘1–2 glasses a day’, ‘0.5 L to 1 L a day’, and ‘More than 1 L a day’. Also in this case individual responses were recoded assigning a score ranging from 0 (low adherence to the MD) to 2 (high adherence to the MD). With regard to water, value 2 was assigned when the response alternative corresponded to ‘More than one liter a day’, value 1 to ‘0.5 L to 1 L a day’ and 0 otherwise. As for other drinks, the MD pyramid guidelines are more generic. For instance, the pyramid recommends ‘Wine in moderation and respecting social beliefs’, without defining precise quantities or gender distinctions. The same is true for beer, although it is not specifically mentioned in the pyramid. In all cases in which the specific food is not included in the pyramid, the scores were assigned following the guidelines for healthy diet formulated by CREA (Center for Research in Agricultural Economics) for the Italian adult population [35]. Accordingly, for wine and beer value 2 was assigned when ‘Rarely’ was the chosen response alternative, value 1 corresponded to ‘1–2 glasses a day’, and value 0 otherwise; whereas for soft drinks the following scores were assigned: 2 corresponding to ‘Zero consumption’, 1 for ‘Seasonal consumption’, and 0 otherwise.

#### 2.3.2. Healthy and Pro-Environmental Behaviors

The latent healthy behaviors were measured considering four indicators related to physical activity (HEALTH 1), weight check (HEALTH 2), smoking behavior (HEALTH 3), and breakfast habits (HEALTH 4) (Table 2). All these can be considered health-enhancing behaviors for a number of reasons. Indeed, physical activity is associated with a decreased risk of cardiovascular disease, diabetes, some types of cancer, and a reduced risk to become overweight or obese [36,37]. The same holds for non-smoking behavior that protects from several diseases and premature death [38,39]. Weight check was included as this behavior is recommended in CREA guidelines for a healthy diet, as it is seen as a way to avoid excess weight gain and health problems related to overweight and obesity [35,40]. Similarly, having breakfast every day is demonstrated to reduce disease risk [41]. All the indicators could assume values ranging from 1 to 3, where 3 characterizes non-smokers who practice physical activity regularly, check their weight, and are used to having breakfast daily.

The latent Variable related to PEB was expressed by four indicators respectively referring to how often consumers pay attention not to throw rubbish on the street (ENV 1), to avoid water (ENV 2) and energy waste (ENV 3), and to recycle (ENV 4). The first three indicators, ranged from 1 (never), to 3 (often), whereas the last one (i.e., recycling) was represented by an index including items related to recycling behavior of paper, glass, plastic, and organic materials respectively. These latter variables were dummies with value 1 indicating ‘recycling’, and value 0 corresponding to ‘not recycling’. The resulting index range was 0–4, where 0 corresponded to individuals that do not have recycling habits and 4 identified people that regularly recycle.

#### 2.3.3. Education and Income

The Italian Household Survey included several questions on respondents’ SES. To the purpose of the survey we only considered questions regarding the level of education and income. The former was assessed through a single question whose response ranged from 1 corresponding to a low level of education (elementary school), to 5 corresponding to a high level of education (EDU 1). As for income, instead, the questionnaire did not include items aimed at explicitly assessing income classes, but comprised several questions geared at indirectly identifying income levels. To the purpose of this analysis we considered three indicators to express the latent income. The former was related to the following question: ‘How would you define your economic situation with respect to last year?’ (INC 1). Answers to this item ranged from 1 (much worsen) to 4 (much improved). The second indicator was referred to a general evaluation of the economic resources of the family, from 1 (insufficient) to 4 (good) (INC 2). Finally, the third indicator was aimed at assessing how much respondents were satisfied with their present economic condition, from 1 (not at all satisfied), to 4 (very satisfied) (INC 3). The values obtained analyzing the correlation between the variables used to measure the income latent construct are the following: corr (INC 1, INC 2) *r* = 0.630 (*p* < 0.01), corr (INC 1, INC 3) *r* = 0.556 (*p* < 0.01), and corr (INC 2, INC 3) *r* = 0.666 (*p* < 0.01). Summary statistics of all items, Mean, Standard Deviation (SD), Minimum (Min) and Maximum (Max) values, are reported in Table 2.

## 3. Results

### 3.1. Sample Size and Characteristics

Table 3 reports the key demographics of the sampled population. Males and females are almost evenly represented and age classes are also homogeneously distributed. Only respondents between 18 and 24 years old are less represented than other age classes. The majority of the sample has a high school diploma, whereas only 10.7% has a post high school education. A considerable percentage of Italians corresponding to more than half of the sampled population (57.8%) has only primary or secondary school education.

The score distribution of the three indexes used to estimate the individual level of adherence to the MD is illustrated in Table 4. With regard to MD1, the majority of the sampled population (48%) has a score ranging from 16 to 20. It is worth noting that 33.4% of the sample has MD1 scores below this range, whilst only 18.4% scored above these values s, which indicates that a significant part of the population follows dietary patterns distant from the MD pyramid guidelines. Opposite evidence emerged with regard to the MD2 fat and salt consumption index. Indeed, almost 65% of the population falls in the score range 5–6, which identifies people who behave in line with the recommended intake for these food categories. Finally, as for the MD3 index the largest percentage of the population (55.1%) follows in the interval 3–5 corresponding to the central values.

### 3.2. Checking Reliability and Validity

Before performing the structural equation model, we verified the reliability (that is, the extent to which the said measurement model is reliable in measuring the intended latent constructs) and the validity (that is, the ability of instrument to measure what it supposed to measure for a latent construct). There are three criteria for assessing the reliability of a measurement model: internal consistency, composed reliability, and average variance extracted (AVE). We tested the internal consistency of the measurement scales using Cronbach’s alpha. The minimum recommended cut-off score is 0.7 [31]. As reported in Table 1, all the elicited Cronbach’s alpha were higher than the cut-off value. Composed reliability allows checking the internal consistency of the indicators used to measure that latent concept. For all indicators, we obtained composed reliability values higher than 0.7 in line with the predetermined composed reliability criteria [42,43]. Finally, AVE represents the average percentage of variance explained by the items in a construct (minimum AVE cutoff ≥ of 0.5) (Table 5).

As for validity, there are two measures required for each measurement model: the convergent validity and the discriminant validity. The convergent validity is achieved when all items in a measurement model are statistically significant and the value of AVE are greater or equal to 0.5. The discriminant validity, represents the extent to which measures of theoretically unrelated constructs do not correlate with one another. The square root of AVE for the construct should be higher than the correlation between the respective construct [44].

### 3.3. Model Testing and Main Results

There are several fit indices to consider in structural equation modeling. In this paper, we took into account the comparative fit index (CFI), the Tucker–Lewis index (TLI), and the root mean square error of approximation (RMSEA). The values obtained respectively for CFI (0.89), TLI (0.91), and RMSEA (0.042) indicate an adequate model fit. 

**Figure 1:**
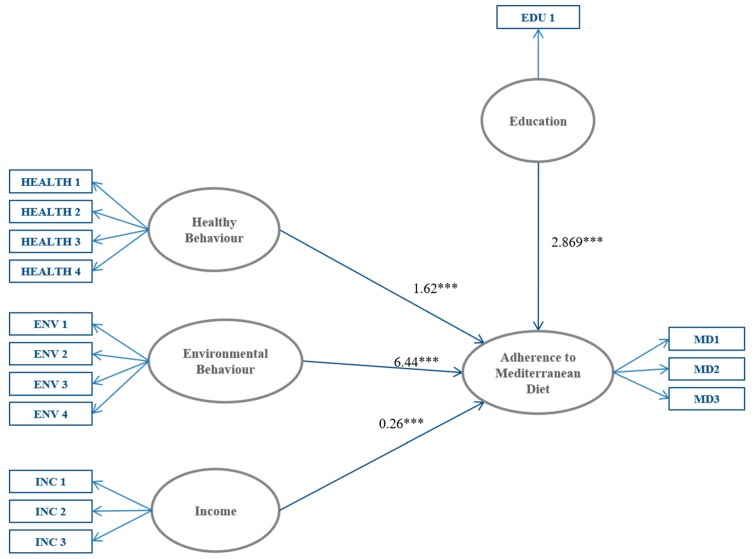
Structural equation model between healthy behaviors, environmental behaviors, income education, and the adherence to Mediterranean diet. Notes: Ovals represent the latent variables, whilst measured variables are indicated in rectangles. The path coefficients of each arrow can be interpreted as common regression weights. *** indicate significance at 0.001 level. MD1: food consumption; MD2: fat and salt consumption; MD3: drink consumption; HEALTH 1: physical activity; HEALTH 2: weight check; HEALTH 3: smoking behavior; HEALTH 4: breakfast habit; ENV 1: attention not to throw rubbish on the street; ENV 2: attention not to waste water; ENV 3: attention not to waste energy; ENV 4: attention to recycling; INC 1: comparison with the economic condition of the previous year; INC 2: evaluation of the household economic condition.

The results of the SEM are illustrated in Figure 1, where arrows represent the relationships between latent constructs. The path coefficients show that hypothesis H1 (i.e., There is a positive relationship between HB and individual level of adherence to the MD) is confirmed. As illustrated by the arrow, the HB latent construct is positively related to MD adherence (1.62, CR = 13.51) (CR: critical ratio). This result suggests that individuals who usually engage in HB are more likely to show dietary patterns in line with the MD recommendations compared to less health-engaged individuals. The results show that also H2 (i.e., There is a positive relationship between PEB and individual level of adherence to the MD) is supported. The arrow direction indicates that PEB positively affect the level of adherence to the MD, and the coefficient magnitude shows that the effect of PEB on MD is stronger than that exerted by HB (6.44, CR = 3.46).

As expected, the model estimates indicate that also individual SES plays a key role in determining the level of adherence to MD. In detail, people with higher income (0.26, CR = 9.28) and education (2.86, CR = 16.87) are more likely to observe the MD model. These results respectively confirm H3 and H4. However, it is worth underlining that the effect of education on the dependent latent MD construct is stronger that that observed with regard to income.

## 4. Discussion

Based on recent data collected through the Italian Household Survey, this study explores the relationship between some aspects of individual lifestyle (i.e., healthy and pro-environmental behaviors), socio-economic status (i.e., income and education), and the level of adherence to the MD.

The results of the SEM outline that individuals who mostly engage in HB and PEB show higher levels of adherence to the MD. In detail, according to the results both HB and PEB are able to drive the extent to which individuals observe the MD pyramid guidelines. This seems to indicate that people’s decision to follow the MD model is not only determined by a mere nutritional need, but is the result of a more complex reasoning which involves their attitudes towards health and the environmental issue. HB and PEB have a common denominator, future orientation, that is the individual tendency to attach more importance to future events than to present satisfaction [23,25]. Future orientation leads individuals to weigh the possible consequences of present actions and to behave in order to avoid any distant negative outcome. As such, it is reasonable to assume that these individuals perceive the MD as a concrete way to address both the health and the environmental issues. Indeed, on the one hand, following the MD is demonstrated to have beneficial effects on health and a preventive effect against disease risk. On the other hand, the plant-centered MD model, which admits only limited amounts of animal based food, represents a way to reduce the environmental impact associated with food production and consumption. It is worth noting that the results reveal a considerable difference in the relationships of HB and PEB with MD. Indeed, PEB have a remarkably stronger effect on the MD adherence compared to HB. This may be motivated by the fact that people’s sense of responsibility towards the environmental issue, which is typically high in those who engage in PEB, may be stronger than individual sense of responsibility towards their own health. However, to establish the reason of such difference in the coefficient magnitudes is out of the scope of this analysis and further research on this topic is needed.

Another relevant aspect emerging from the analysis concerns the role of SES. Indeed, both income and education are positively related to the level of adherence to the MD. Previous works demonstrated that healthy eating, based on high consumption of low-energy/nutrient-dense foods (i.e., fruits and vegetables) and low consumption of nutrient-empty/energy-dense foods, increases the costs associated with food consumption. This creates an obstacle for individuals in following a plant-based diet such as the MD [7]. The role of education in influencing the degree of adherence to MD is even stronger. The relationship of education with MD may find explanation in the well-known mediating role of nutritional knowledge. There is evidence that the longer the education, the higher the nutritional knowledge and the individual awareness concerning food related issues. This leads individuals to be more keened to follow balanced dietary patterns [13,45]. Additionally, previous works highlighted that high education is also positively associated with environmental attitudes and increased pro-environmental behaviors [21]. As such, it is reasonable to assume that highly educated individuals are more likely to follow the MD as a sustainable dietary pattern. Overall, the results related to SES are in line with previous findings highlighting that income and education are independently and strongly associated with a higher adherence to MD [13].

These results contribute to extend current evidence concerning the main determinants of individual adherence to MD and provide novel insights for future policy interventions geared at promoting this dietary model. However, this work has some limitations that need to be acknowledged. Firstly, the consumption frequency data are based on self-reported intakes and, consequently, they may suffer from bias (e.g., under-/over- estimation, social desirability bias). Same for the self-reported data on income and education. A second issue regards the questions used to construct our MD1, MD2, and MD3 indexes. Indeed, the Italian Household Survey is not specifically aimed at collecting food consumption data. As such, the questions are sometimes too general to elicit specific intake differences within the same food group (e.g., whole and refined grains) or to capture differences in how foods are cooked (e.g., fried, steamed, etc.). As such, the scoring of individual responses may be biased. Moreover, the survey lacks specific questions related to certain aspects of individual lifestyle such as conviviality and cultural elements that have been recently incorporated into the MD pyramid. Despite these limitations however, it is important that the study is based on a large sample size, representative of the Italian population as a Mediterranean country, which allows us to generalize the results.

## 5. Conclusions

The main findings of this study reveal that MD adherence is determined by a number of contributing factors. The MD model does not only represent a healthy way of eating, but is a complementary part of individual lifestyle together with health and pro-environmental attitudes and behaviors. Indeed, the MD seems to be part of a sustainability-oriented way of thinking and acting and, to be effective, future policy interventions should be able to emphasize this aspect. In other words, policy measures geared at promoting the MD should be focused not only on the mere nutritional aspects, but also on the sustainable, cultural, and social dimensions that unequivocally characterize the MD heritage. Furthermore, future policy strategies aimed at contrasting the gradual disappearance of the MD heritage in Mediterranean countries should consider the role of income and education in affecting diet quality. Indeed, from a public health perspective, reducing SES disparities among different population segments may result in nutritional improvements and decreased environmental impact.

Further research on the main determinants of individual adherence to the MD would make a significant contribution to extend current evidence on this issue. Future studies should employ an ad hoc questionnaire with more precise food intake questions that could allow, for instance, to distinguish between gender-specific nutritional needs. Moreover, additional aspects related to pro-environmental and healthy behaviors should be explored, focusing not only on the mere actions, but investigating the reasons that lead individuals to act in a certain way.

## Figures and Tables

**Table 1:** Food items in the MD1 index, related recommended intake and scoring criterion.

Index	Food Items	Recommended Intake	Scoring Criterion	
			Response Alternative	Assigned Score
**MD1**	Cereals (rice, pasta, bread, etc.)	1–2 portion(s) every main meal	Never	0
			Less than once a week	0
			Sometimes in a week	0
	Leaf vegetables cooked and raw (spinach, salad, etc.)	≥2 portions every main meal	Once a day	1
	Other vegetables (fennel, tomato, pepper, artichokes, etc.)	≥2 portions every main meal	More than once a day	2
	Fruit	1–2 portion(s) every main meal		
	Legumes	≥2 portions weekly	Never	0
			Less than once a week	0
			Sometimes in a week	2
			Once a day	1
	Potatoes	≤3 portions weekly	More than once a day	0
	Fish	≥2 portions weekly	Never	0
			Less than once a week	1
			Sometimes in a week	2
			Once a day	0
			More than once a day	0
	Processed meat	≤1 portions weekly	Never	0
	Red meat (beef)	<2 portions weekly	Less than once a week	2
	Pork meat	<2 portions weekly	Sometimes in a week	1
	Sweets	<2 portions weekly	Once a day	0
	Salty snacks	<2 portions weekly	More than once a day	0
	White meat (turkey, chicken, rabbit, veal, etc.)	2 portions weekly	Never	0
			Less than once a week	1
			Sometimes in a week	2
			Once a day	0
	Eggs	2–4 portions weekly	More than once a day	0
	Milk	2 portions daily	Never	0
			Less than once a week	0
			Sometimes in a week	0
			Once a day	2
			More than once a day	1
	Dairy products and cheeses	2 portions daily		

Note: p = portion(s). Serving size is based on frugality, as indicated in the Mediterranean diet (MD) pyramid [33,34]. MD1: food consumption index.

**Table 2:** Proposed item measures.

Dimension	Item	Items Description	Mean	SD	Min	Max
Adherence to MD (*α* = 0.76)	MD1	Food consumption	17.01	3.83	0	29
	MD2	Fat and salt consumption	4.91	1.01	0	6
	MD3	Drink consumption	3.51	1.73	0	8
Healthy behaviors (*α* = 0.82)	HEALTH 1	Physical activity	1.53	0.83	1	3
	HEALTH 2	Weight check	1.72	0.79	1	3
	HEALTH 3	Smoking behavior	2.36	0.80	1	3
	HEALTH 4	Breakfast habit	1.89	0.50	1	3
Pro-environmental behaviors (*α* = 0.89)	ENV 1	Attention not to throw rubbish on the street	1.42	0.73	1	3
	ENV 2	Attention not to waste water	2.64	0.59	1	3
	ENV 3	Attention not to waste energy	1.77	0.82	1	3
	ENV 4	Attention to recycling	3.38	1.25	0	4
Income (*α* = 0.78)	INC 1	Comparison with the economic condition of the previous year	2.28	0.72	1	4
	INC 2	Evaluation of the household economic condition	2.47	0.64	1	4
	INC 3	Satisfaction for the personal economic condition	2.31	0.77	1	4
Education	EDU 1	Level of education	2.33	1.16	1	5

Note: SD = Standard Deviation.

**Table 3:** Sample characteristics.

Socio-Demographic Characteristics	
	% of total (*n* = 42,000)
Gender	
Male	51.2
Female	48.8
Age	
18–24	9.2
25–34	13.5
35–44	17.6
45–54	17.6
55–64	15.6
65–74	13.4
>75	13.1
Education	
Primary school	28.4
Secondary school	29.4
High school	31.5
Degree	2.2
Master degree	8.5

**Table 4:** MD1, MD2, and MD3 score distribution.

Index Scores	% of Total (*n* = 42,000)
MD1—Food consumption index	
1–5	0.3
6–10	4.8
11–15	28.3
16–20	48.3
21–25	17.7
>25	0.7
MD2—Fat & salt consumption index	
1–2	2.2
3–4	33.1
5–6	64.4
MD3—Drink consumption index	
0–2	30.9
3–5	55.1
6–8	14.0

**Table 5:** Reliability measures.

Latent construct	Cronbachs Alpha	Composite Reliability	AVE
Adherence to MD	0.76	0.88	0.79
Healthy behaviors	0.82	0.87	0.80
Pro-environmental behaviors	0.89	0.88	0.69
Income	0.78	0.76	0.82

AVE: average variance extracted.
